# The Two Faces of Nematode Infection: Virulence and Immunomodulatory Molecules From Nematode Parasites of Mammals, Insects and Plants

**DOI:** 10.3389/fmicb.2020.577846

**Published:** 2020-12-02

**Authors:** Sarah D. Bobardt, Adler R. Dillman, Meera G. Nair

**Affiliations:** ^1^Division of Biomedical Sciences, School of Medicine, University of California, Riverside, Riverside, CA, United States; ^2^Department of Nematology, University of California, Riverside, Riverside, CA, United States

**Keywords:** entomopathogenic nematode, inflammatory disorders, vaccination, excretory and secretory products, soil-transmitted helminth

## Abstract

Helminths stage a powerful infection that allows the parasite to damage host tissue through migration and feeding while simultaneously evading the host immune system. This feat is accomplished in part through the release of a diverse set of molecules that contribute to pathogenicity and immune suppression. Many of these molecules have been characterized in terms of their ability to influence the infectious capabilities of helminths across the tree of life. These include nematodes that infect insects, known as entomopathogenic nematodes (EPN) and plants with applications in agriculture and medicine. In this review we will first discuss the nematode virulence factors, which aid parasite colonization or tissue invasion, and cause many of the negative symptoms associated with infection. These include enzymes involved in detoxification, factors essential for parasite development and growth, and highly immunogenic ES proteins. We also explore how these parasites use several classes of molecules (proteins, carbohydrates, and nucleic acids) to evade the host’s immune defenses. For example, helminths release immunomodulatory molecules in extracellular vesicles that may be protective in allergy and inflammatory disease. Collectively, these nematode-derived molecules allow parasites to persist for months or even years in a host, avoiding being killed or expelled by the immune system. Here, we evaluate these molecules, for their individual and combined potential as vaccine candidates, targets for anthelminthic drugs, and therapeutics for allergy and inflammatory disease. Last, we evaluate shared virulence and immunomodulatory mechanisms between mammalian and non-mammalian plant parasitic nematodes and EPNs, and discuss the utility of EPNs as a cost-effective model for studying nematode-derived molecules. Better knowledge of the virulence and immunomodulatory molecules from both entomopathogenic nematodes and soil-based helminths will allow for their use as beneficial agents in fighting disease and pests, divorced from their pathogenic consequences.

## Introduction

Parasitic nematodes infect hosts from almost all branches of the tree of life, often using conserved strategies to successfully invade host tissue while evading the rapid immune response against them (Blaxter and Koutsovoulos, 2015). Their ability to manipulate host immunity is incredible. Consider a 21-year-old woman who presents with no pathology, inflammation, or any symptoms other than the sensation of something moving in her eye (Shah and Saldana, 2010). On examination, a live two cm nematode was found in the superior subconjunctival space of her eye and removed. Subsequent blood testing revealed microfilaremia (i.e., the presence of thousands of juvenile parasitic nematodes in her blood and likely other tissues). Her medical history revealed that she had picked up this infection on a trip to Nigeria 6 years earlier. This carefully coordinated infection is facilitated in part through immunomodulatory excretory/secretory (ES) products that allow the parasite to establish long-lived infection. Given their potent immunomodulatory properties, understanding these nematode-derived products may lead to the development of new anthelminthics and vaccines to overcome host immune suppression, or instead exploited for new therapies in allergy and inflammatory diseases.

Nematode-derived products comprise a diverse range of molecules, including proteins, lipids, carbohydrates, and nucleic acids (Maizels et al., 2018). They have a multitude of effects on the host, from toxic virulence factors that cause tissue damage to powerful immunomodulators. Some of these molecules are homologous to host molecules, allowing the parasite to manipulate immune cell function by mimicking host proteins or producing miRNAs that target host gene expression (Buck et al., 2014; Johnston et al., 2017). Others are unique to parasites and are not found in the host (Periago and Bethony, 2012; Flynn et al., 2019). These virulence factors are important for the parasite’s infectivity and growth within the host, and can cause host tissue damage.

Entomopathogenic nematodes (EPN), which infect and kill their insect hosts within days, are currently used for controlling agricultural pests (Cooper and Eleftherianos, 2016). These parasites also provide a valuable model of parasitic nematode infections with significantly lower costs than rodent models. EPNs produce venom proteins with significant similarity to many proteins found in mammalian pathogenic nematodes (Lu et al., 2017; Chang et al., 2019). EPNs, like nematodes that infect mammals, need to suppress and/or evade the initial immune response (Bai et al., 2013; Brivio and Mastore, 2018). For this reason, identification of EPN-derived molecules and their effects on the host may be translational to nematode parasites of mammals.

Several recent reviews have provided significant insight into the immunomodulatory role of nematode-derived molecules, in particular excreted and secreted proteins (McSorley et al., 2013; Gazzinelli-Guimaraes and Nutman, 2018; Maizels et al., 2018; Zakeri et al., 2018; Maizels, 2020; Ryan et al., 2020). In this review, we highlight recently characterized nematode-derived molecules involved in host-parasite interactions, expand our discussion to non-protein molecules (e.g., lipids, nucleic acids) as potential for therapeutic targets, and investigate the utility of non-mammalian model systems (e.g., insect, plant) to understand host-nematode interactions. We first describe nematode virulence factors, how they mediate host tissue damage, and their utility as anthelminthic or vaccine targets. Next, we discuss nematode-derived products that suppress the host immune response and can be harnessed therapeutically for their immunomodulatory properties. Finally, we evaluate what can be learned from EPNs and plant-parasitic nematodes (PPNs) as models for mammalian-pathogenic nematodes to identify new parasite virulence and immunomodulatory molecules for immunotherapies and drug targets. Studying nematode-derived products’ role in host-parasite interactions provides valuable insight for novel treatments to fight off infection or alleviate allergic and inflammatory diseases.

## Virulence Factors

**FIGURE 1:**
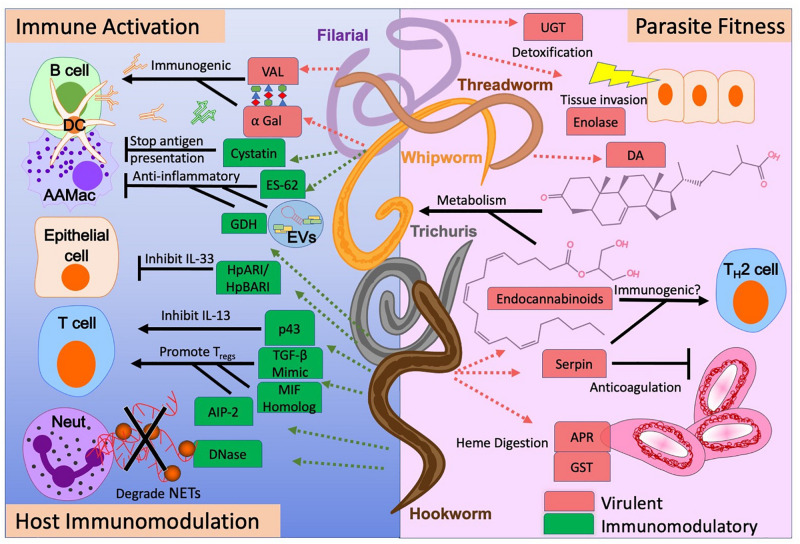
The pleiotropic functions of nematode-derived molecules. Functions range from promoting or inhibiting the host immune response (left), to providing essential physiologic functions for the nematode parasite (right). Understanding the virulence (red) and immunomodulatory (green) potential for specific nematode-derived molecules allows us to determine their utility as vaccines, anthelminthics, or new therapeutics for allergic or inflammatory diseases.

Many parasitic nematodes transition to parasitism from a developmentally arrested lifecycle stage in order to obtain resources, complete their lifecycle, and find a long-lasting niche (Hotez et al., 2008). To this end, they can produce a wide variety of molecules to assist in their ability to attack, invade, and digest host tissue (Periago and Bethony, 2012). Their arsenal of host virulence molecules can be targeted to develop vaccines or design anthelminthics to reduce worm burden and mitigate host pathology in infected individuals. Significant research on anthelminthics and vaccines is performed in preclinical rodent models to find potential targets for vaccine development prior to the initiation of human research and clinical trials (Table 1 and Figure 1). Here, we highlight promising nematode-derived molecules for anthelminthic and vaccine targets. These include enzymes involved in host tissue invasion and parasite feeding, nematode-derived molecules necessary for parasite development, and immunogenic ES proteins as vaccine candidates.

**TABLE 1:** Nematode-derived molecules and their potential for therapeutic use as vaccine, anthelminthic or anti-inflammatory treatments.

	Therapeutic potential	Name	Function
Preclinical	Anthelminthic/Vaccine	UDP-glucuronosyltransferase (UGT)	Detoxifies molecules Saeed et al., 2018
		Enolase	Binds to plasminogen and assists in invading host tissue Jiang et al., 2019
		Endocannabinoid (eCB)	Regulates parasite metabolism and development Batugedara et al., 2018; Ma et al., 2019b
		Dafachronic acid (DA)	
		Serpin	Inhibits host blood coagulation Yi et al., 2010
		α-Gal	Induces host production of protective antibodies Hodžić et al., 2020

Clinical		Hemoglobinase aspartic protease (APR-1)	Involved in the hemoglobin detoxification pathway Hotez et al., 2013
		Glutathione S-transferase (GST-1)	
		Ancylostoma secreted protein-2 (ASP-2)	Although effective in animal models, caused generalized urticaria in clinical trial Diemert et al., 2012

Preclinical	Anti-Inflammatory	Anti-inflammatory protein-2 (AIP)	Promotes Tregs to suppress airway inflammation Navarro et al., 2016
		Alarmin release inhibitor (ARI)	Binds to IL-33 and inhibits its release Osbourn et al., 2017
		Binds ARI (BARI)	Binds to receptor for IL-33 Vacca et al., 2020
		Glutamate dehydrogenase (GDH)	Induces anti-inflammatory eicosanoid switch de Los Reyes et al., 2020
		Cystatin	Reduces inflammatory cytokines and promotes Tregs Hartmann et al., 2002
		Macrophage inhibitory factor (MIF)	Suppresses the immune system through structures homologous to host cytokines Johnston et al., 2017; Cho et al., 2011
		TGF-β mimic (TGM)	
		p43	Binds and inhibits IL-13 Bancroft et al., 2019
		ES-62	Interacts with a broad range of immune cells to downregulate an inflammatory response Suckling et al., 2018
		DNase	Impairs worm killing by degrading neutrophil extracellular traps Bouchery et al., 2020
		Extracellular vesicles (EVs)	Disrupt immune cell function and contain miRNAs Eichenberger et al., 2018

### Nematode-Derived Enzymes

Nematodes produce a variety of enzymes for host tissue digestion and feeding that provide useful targets for vaccine development or anthelminthics. For example, enolases are plasminogen-binding surface proteins that are involved in assisting parasites in invading host tissue by promoting the degradation of fibrin (Ayón-Núñez et al., 2018; Jiang et al., 2019). Vaccination with a *Trichinella spiralis* enolase resulted in the production of specific antibodies in mice, however, this did not lead to a striking reduction in worm burden (Zhang et al., 2018). Additionally, parasite-derived enolases were found in the serum of *Brugia malayi*-infected individuals, indicating its potential as a diagnostic molecule (Reamtong et al., 2019).

For hookworms, which feed on the blood of their host causing anemia, enzymes involved in processing blood hemoglobin have demonstrated promising vaccine potential (Periago and Bethony, 2012; Hotez et al., 2013). Specifically, the *N. americanus* hemoglobinase aspartic protease (Na-APR-1) and the heme detoxifying Na-Glutathione S-transferase (Na-GST-1), are currently in clinical trials for a hookworm vaccine, with early results showing the vaccine is safe and immunogenic (Zhan et al., 2010; Bottazzi, 2013, 2017; Diemert et al., 2017). Several phase 1 clinical trials have been completed with recombinant Na-APR-1 vaccine (Bottazzi, 2013). Administration of the recombinant Na-GST-1 vaccine to hookworm-naïve individuals as well as those from hookworm endemic regions in Brazil was found to be safe and immunogenic, and phase 2 clinical trials are underway (Bottazzi, 2017). The success of these trials suggest that safe and effective vaccines could be developed by targeting enzymes integral to nematode feeding and survival. For example, in filarial nematodes, enzymes have provided promising targets for anthelminthic and vaccine development. These include GST, which protects the nematode parasite by neutralizing host cytotoxic products (e.g., reactive oxygen species) and mediating drug resistance, and UDP-glucuronosyltransferase (Bm-UGT), a detoxifying enzyme expressed in the *B. malayi* intestinal lumen that was essential for its survival (Saeed et al., 2018; Flynn et al., 2019).

### Nematode-Derived Molecules Involved in Growth and Metabolism

Lipid-derived molecules are involved in a variety of biological functions in parasitic nematodes including metabolism and development, making them of particular interest as potential novel anthelminthics that target parasite fitness and development (Ma et al., 2020). Endocannabinoids are lipid-derived molecules important for metabolic homeostasis and immunity, among other functions, and are produced by both mammals and nematodes (Batugedara et al., 2018). The specific function of parasitic nematode-derived endocannabinoids in the host is unclear. However, functional studies for endocannabinoids have been possible in the free-living nematode *C. elegans*, where endocannabinoids, 2-arachidonoyl glycerol and anandamide played a significant role in metabolism and aversion to pain (Oakes et al., 2017; Galles et al., 2018). Understanding the interplay between host and parasitic nematode-derived endocannabinoids might reveal new immune and metabolic targets to reduce parasite fitness or improve the host response.

The steroidal hormone dafachronic acid (DA) modulates nematode lipid metabolism and development, and the ligand-binding domain for the steroid hormone nuclear receptor for DA (DAF-12) is highly conserved among nematode species, such as *C. elegans, N. brasiliensis, Haemonchus contortus*, and *Strongyloides stercoralis*, therefore targeting these receptors may have therapeutic potential to impair parasite fitness (Ogawa et al., 2009; Patton et al., 2018; Ma et al., 2019a,b; Ayoade et al., 2020). In *C. elegans*, the DAF-12 system acts to inhibit dauer formation, and in *H. contortus*, DA promoted transition from free-living to parasitism by modulating dauer-like signaling (Ogawa et al., 2009; Ma et al., 2019a,b). Recent investigation of the DAF-12 system in *S. stercoralis* hyperinfection supports the therapeutic potential of inducing this steroid hormone pathway. In a mouse model of *S. stercoralis* hyperinfection, which is an often fatal condition in immunocompromised individuals, DA treatment was protective and reduced *S. stercoralis* parasite burdens by suppressing the development of autoinfective L3a larvae (Patton et al., 2018).

### Immunogenic Nematode ES Proteins

Molecules integral to the parasite’s growth and ability to colonize and feed on the host offer promising vaccine and anthelminthic targets. However other weapons of warfare employed by the worms, such as excreted molecules that are immunogenic and promote a protective anti-helminthic immune response can also be considered as vaccines or adjuvants.

The family of venom allergen-like proteins (VAL) family is one such example. This family has been extensively studied especially given their high expression in many parasitic nematodes (Wilbers et al., 2018). While these proteins are conserved among a wide variety of nematodes, their functions are diverse—including examples of both pro-inflammatory and immunosuppressive molecules. Here we explore some of the most pertinent examples from this family, focusing on the VAL proteins that have demonstrated immunogenic properties for vaccine potential. VAL proteins are homologs of vespid (wasp) venom proteins, the latter of which are locally toxic and can induce allergic and inflammatory responses in humans (King and Valentine, 1987; Tawe et al., 2000; Murray et al., 2001). This makes nematode-derived homologs of these proteins of particular interest in understanding host-nematode interactions that lead to the excessive pathology for the host (King and Valentine, 1987; Wilbers et al., 2018; Tawe et al., 2000; Murray et al., 2001). VAL proteins are conserved in several parasitic nematodes, including *Heligmosomoides polygyrus*, *B. malayi*, *Trichinella pseudospiralis*, and *Teladorsagia circumcincta* (Ellis et al., 2014; Wang et al., 2017a; Asojo et al., 2018; Darwiche et al., 2018; Wilbers et al., 2018). Notably, VALs are highly expressed: a study of the secreted products from the gastrointestinal murine parasite *H. polygyrus* revealed that members of the VAL protein family were the most abundant product (Hewitson et al., 2011). Due to their abundance and conserved structure, VALs have been considered as vaccine candidates. For instance, the *B. malayi* protein Bm-VAL-1 is highly immunogenic, promoting antibody and T cell responses in humans, and conferring protection in vaccination models in mice and jirds (Maizels et al., 1995; Kalyanasundaram and Balumuri, 2011; Darwiche et al., 2018). To target larval stages, Bm-VAL-1 and *B. malayi* abundant larval transcript-2 (Bm-ALT-2) were combined in a multivalent vaccine, which successfully increased antibody titers and provided enhanced worm killing in a challenge infection in mice (Kalyanasundaram and Balumuri, 2011; Anugraha et al., 2013). The importance of combining antigens as a vaccine strategy for a more effective immune response is increasingly being recognized. In *H. polygyrus* infection, immunization with a cocktail of three *H. polygyrus* VALs induced antibody production that protected mice from challenge *H. polygyrus* infection (Hewitson et al., 2015). The biological function of VALs in infection is unclear, however, given that they are secreted sterol-binding proteins, they may bind immunomodulatory molecules such as prostaglandins and leukotrienes, which have both immune stimulatory and regulatory roles (Honda and Kabashima, 2019).

The immunogenic properties of VALs have made them potential targets for vaccine development. Na-ASP-2 (Ancylostoma-secreted protein), another member of the VAL family, initially showed promise as a hookworm vaccine (Bethony et al., 2005; Diemert et al., 2012). This allergen-like protein was associated with the production of IgE and IgG4 antibody responses that correlate with reduced risk of high *Necator americanus* burdens in endemically affected areas. Further, validation studies in dogs confirmed that Na-ASP-2 specific antibodies were protective in challenge infections (Bethony et al., 2005). However, early clinical trials resulted in generalized urticarial reactions in many individuals, associated with pre-existing Na-ASP-2-specific IgE (Diemert et al., 2012). This failed clinical trial is a cautionary tale for vaccine development against parasitic nematodes. First, the potential for non-protective allergic-immune responses in previously exposed individuals in endemic areas needs to be considered. Additionally, anti-inflammatory nematode-derived molecules that are necessary to mitigate host tissue damage and inflammation may need to be carefully considered before being used as vaccine or therapeutic targets, since inhibiting these may be more pathogenic than beneficial for the host. Although the effectiveness of VALs as vaccine targets for helminths is challenged by these recent studies, their immunogenic potential may be harnessed for use as adjuvants against other infectious pathogens. For example, recombinant ASP-1 derived from the filarial nematode *Onchocerca volvulus*, Ov-ASP-1, has shown promise as an adjuvant for vaccines against viral infections, such as HIV, SARS-CoV, and influenza, augmenting viral antigen-specific antibody titers in immunization studies in mice (MacDonald et al., 2005).

Serine protease inhibitors (serpins) constitute another highly conserved family of nematode ES proteins, identified in many nematodes, including *B. malayi*, *Anisakis simplex*, and *H. contortus* (Zang et al., 2000; Bennuru et al., 2009; Yi et al., 2010; Valdivieso et al., 2015). *In vitro* studies of a serpin derived from *H. contortus* showed that it reduced blood coagulation (Yi et al., 2010). The anti-coagulation function of serpins is likely an important feeding mechanism for blood-feeding nematodes. *B. malayi* microfilariae secrete serpins, perhaps to mitigate a coagulation response to excess circulating microfilariae in the bloodstream during chronic infection (Zang et al., 2000). *B. malayi* serpins are also immunogenic and stimulate mouse and human B and T cell responses, however, this immune response is short-lived, suggesting that serpins alone are not effective vaccine candidates for long-term immunity (Zang et al., 2000). It is interesting to note that despite many preclinical studies on nematode-secreted proteins, only nematode enzymes are currently the subject of ongoing vaccine clinical trials (Table 1). This suggests that targeting virulence factors, which are integral components of the worm’s physiology may offer the most promising vaccination targets.

## Immunomodulatory Molecules

Parasitic nematodes have evolved multiple mechanisms to evade the host immune system, allowing persistence in their host, in some cases for decades, without being killed (Maizels et al., 2018). Nematode-derived products are key to the immunomodulatory capabilities of the parasites, and investigating their mechanism of action may identify novel therapies for allergic and inflammatory diseases. Reviewing current research into which molecules show the most promise for the development of immunotherapies is an ongoing conversation that has already received a significant amount of attention (Smallwood et al., 2017; Maizels et al., 2018; Bohnacker et al., 2020; Coakley and Harris, 2020; Ryan et al., 2020; White et al., 2020). Here we contribute to that conversation by contextualizing the most current advances in our understanding of immunomodulatory capabilities of nematodes and identifying the molecules that appear to show the most promise for further research.

Identifying the specific molecules within parasitic nematode ES or extract that have the strongest immunomodulatory potential is a main focus in the field of “helminth therapeutics” (Ryan et al., 2020). Nematode-derived immunomodulatory molecules include mimics of host immune mediators as well as novel molecules that are unique to parasites themselves. Many of the products with extensive characterization are proteins. However non-proteins products, such as carbohydrates and small RNAs, are currently being studied for their potential role in immunomodulation (Prasanphanich et al., 2013; Hokke and van Diepen, 2017; Maizels et al., 2018). Mechanisms of immunomodulation for the main nematode-derived molecules discussed here are summarized in Figure 1.

### Glycans

The differential glycosylation of lipids and proteins during the lifecycle of parasitic nematodes provides a unique opportunity for the development of vaccines and novel anthelminthics (Prasanphanich et al., 2013; Hokke and van Diepen, 2017).

Glycosylation patterns unique to the parasite are potential vaccine targets, because they are distinct from host glycosylation patterns and potentially more immunogenic, acting as pathogen-associated molecular patterns. On the other hand, glycosylation patterns that mimic the host can be explored for their immunomodulatory potential, providing novel immunotherapies. Gala1–3GalNAc-R (α-Gal), a parasite-specific glycan epitope produced by the sheep pathogen *H. contortus* induced an IgG response in lambs and is implicated in protection against *H. contortus* challenge infections (van Stijn et al., 2010). Similarly, glycosylation patterns are essential for the host to recognize the glycans on the surfaces of mucin-like proteins expressed by *T. canis*, and led to pro-inflammatory cytokine expression by human THP-1 macrophages (Długosz et al., 2019).

In the anaphylactic reaction known as α-Gal Syndrome (AGS), humans produce IgE in response to α-Gal present in red meat. However, humans infected with *T. canis* had reduced IgE antibodies to α-Gal caps on N-glycans, indicating that the parasites may be able to downregulate the allergic response, even though this is not an epitope that the worms themselves make (Hodžić et al., 2020). The ability of the parasite to suppress the immune response to oligosaccharides provides evidence for the “hygiene hypothesis” which argues that an increased sensitivity to a wide variety of allergens may result from a reduction in helminth infections in countries with stronger sanitation infrastructure (Yazdanbakhsh et al., 2002). In another example of immunomodulation, N-glycans produced by the canine heartworm *Dirofilaria immitis* allowed the worm to hide from the host immune system, by imitating host glycosylation patterns and also using unique glycosylation patterns that interfered with host binding to other nematode-derived molecules, a technique known as glycol-gimmickry (Martini et al., 2019). Further, changing the glycosylation patterns on proteins in *H. polygyrus* resulted in an increase in proinflammatory cytokines and a decrease in nematode-specific IgG1 in Balb/c mice (Doligalska et al., 2013). Together these studies show the importance of glycosylation patterns in both immunogenicity and evasion of the host immune system by parasitic nematodes.

### Proteins

ES proteins have systemic effects on the immune system, which could be harnessed as therapies for allergy and inflammatory diseases. For example, *in vitro* treatment of macrophages with *T. spiralis* ES generated a regulatory phenotype that prevented airway allergic inflammation in mice (Kang et al., 2019). Similarly, in the dog hookworm *A. caninum*, the secreted anti-inflammatory protein-2 (AIP-2) suppressed airway inflammation in an asthma model in mice in a dendritic cell and Treg-dependent pathway (Navarro et al., 2016). The *H. polygyrus* ES proteins, *H. polygyrus* Alarmin Release Inhibitor (HpARI) and *H. polygyrus* Binds Alarmin Receptor and Inhibits (HpBARI), were identified due to their ability to downregulate the initiation of both type 2 allergic and parasitic responses through the IL-33-ST2 pathway. HpARI bound to the alarmin IL-33 in necrotic cells and prevented its release, while HpBARI binds IL-33 receptor ST2, preventing IL-33 engagement (Osbourn et al., 2017; Vacca et al., 2020). Intranasal administration of HpARI followed by infection with the skin-penetrating *Nippostrongylus brasiliensis* lead to greater intestinal worm burdens in comparison to untreated infected mice, indicating that a *H. polygryus*-specific product could impair immune responses to a different but related parasitic worm. HpBARI administration suppressed Th2 inflammatory responses to the extract from the allergenic fungus *Alternaria*. While *H. polygyrus* is a nematode parasite of mice, and HpBARI targets murine ST2, a homolog of HpBARI (HpBARI_Hom2), was identified that could effectively suppress the human ST2, supporting the clinical relevance of these findings. A similar strategy to inhibit Th2 cytokine responses is employed by *T. muris* with p43, the most abundant protein in its excretome/secretome (Bancroft et al., 2019). *T. muris* p43 contains structural domains homologous to thrombospondin and the IL-13 receptor, which allowed it to tether to matrix proteoglycans and bind and inhibit IL-13. Functionally, p43 inhibits its function in promoting worm expulsion. Another recently identified candidate for immune modulation is the enzyme *H. polygyrus*-derived *Hpb* glutamate dehydrogenase (GDH), which reduced allergic airway inflammation in mice by inducing a switch from pro-inflammatory to anti-inflammatory eicosanoids (e.g., prostaglandins). *Hpb*-GDH was effective at suppressing inflammatory pathways in both mouse and human macrophages and granulocytes by inhibiting the 5-lipooxygenase and instead promoting the cyclooxygenase pathway leading to the synthesis of prostanoids and the downregulation of 5-LOX metabolites (de Los Reyes et al., 2020). Identifying nematode-derived enzymes that target host immune-metabolic pathways with the resulting effect of suppressing inflammatory responses is an exciting new research avenue that may offer novel immunotherapeutics of allergic diseases.

Nematode-derived cysteine protease inhibitors (cystatins) also have demonstrated anti-inflammatory functions. Cystatin from the filarial nematode *Acanthocheilonema viteae* downregulated Th2 cytokine responses in an airway allergy model in Balb/c mice, including decreased IL-5 and IL-13 in the broncho-alveolar lavage (Daniłowicz-Luebert et al., 2013). In *in vitro* microglial cultures from cells harvested from rat brains and stimulated with LPS, *A. viteae* cystatin downregulated nitric oxide and TNFα expression as well as mRNA expression of the pro-inflammatory cytokines iNOS and COX-2, providing promise for therapies for neurodegenerative diseases, such as Parkinson’s disease (Behrendt et al., 2016). Cystatins from filarial nematode *B. malayi* were also immunosuppressive: treatment with recombinant Bm cystatin was able to reduce dextran sulfate sodium (DSS)-induced colitis in mice (Bisht et al., 2019). Specifically, Bm cystatin led to increased Tregs in the colon and alternative activation of peritoneal macrophages. Recently, cystatin from the ES products of the zoonotic nematode *T. spiralis*, rTsCstN, was discovered as structurally homologous to human cystatin (Kobpornchai et al., 2020). Functionally, rTsCstN suppressed inflammatory cytokine production by LPS-treated mouse bone marrow derived macrophages. Cystatins are found in a wide variety of nematode species, including *O. volvulus, H. contortus, and B. malayi* (Hartmann et al., 2002; Hartmann and Lucius, 2003; Gregory and Maizels, 2008; Arumugam et al., 2014; Wang et al., 2017c) and their potential as immunomodulators is being explored.

Cytokines are commonly mimicked by parasitic nematodes in their efforts to modulate the host immune system. One such ortholog is Macrophage Inhibitory Factor (MIF), which is produced by many nematodes including *B. malayi*, *Anisakis simplex*, *Wuchereria bancrofti*, and *H. contortus* (Bennuru et al., 2009; Chauhan et al., 2015; Wang et al., 2017b; Harischandra et al., 2018). Murine MIF is important for alternative activation of macrophages, promotes the Th2 response during nematode infection, and is required for optimal worm clearance (Filbey et al., 2019). On the other hand, nematode MIF homologs appear to have an immunosuppressive effect (Cho et al., 2011). MIF isolated from *A. simplex* increased Treg responses and reduced colitis severity. Here, mice treated with rAs-MIF regained previously lost weight and had lower disease activity indices in DSS-induced colitis. Another modulator of Tregs are TGF-β orthologs which are found in several parasitic nematodes, including *H. polygyrus*, *N. brasiliensis*, and *T. circumcincta* (McSorley et al., 2010). Hp-TGM was recently identified as a TGF-β mimic produced by *H. polygyrus* (Johnston et al., 2017). Interestingly, this mimic is structurally distinct from mammalian TGF-β, however, it is able to bind to mouse and human TGF-β receptors and induce Foxp3 expression in Treg cells. Furthermore, Hp-TGM was immunosuppressive in an allogenic skin graft model where it delayed graft rejection.

Across the proteomes of parasitic nematodes, there is consistency in the presence of amino acid motifs recognizable by T-cell receptors, known as T-cell-exposed motifs or TCEMs (Homan and Bremel, 2018). Using bioinformatics to analyze the proteomes of a wide variety of nematodes, many proteins have been identified with extremely high indices of predicted immunosuppression, indicating that the protein is likely to promote Treg responses. For instance, the hookworm, *A. ceylanicum* alone had over 500 peptides with a highly suppressive index (Homan and Bremel, 2018). Given its rapidity and cost-effectiveness, the ability to screen *in silico* for immunotherapeutic nematode-derived proteins may constitute an important frontier for research in nematode immunomodulation.

Some products have an effect on a wide range of immune cells. These include ES-62, a secreted protein from the nematode *A. viteae*, which interacts with B cells, dendritic cells, macrophages, and mast cells to downregulate inflammatory responses (Pineda et al., 2014). This protein’s anti-inflammatory potential is reliant on post-translational modification including the attachment of phosphorlycholines. Current research on characterizing small molecule analogs for ES-62 is an example of the potential for nematode-derived products to be the impetus for the development of immunotherapies (Suckling et al., 2018).

A recent study highlighted nematode-secreted DNases as a novel mechanism for impairing neutrophil-mediated killing (Bouchery et al., 2020). Within hours post-infection with rat hookworm *N. brasiliensis*, host neutrophils swarmed invading nematodes and released neutrophil extracellular traps (NETs) comprised of nucleic acids, histones, and granular proteins. This provides evidence that NETs, originally identified in bacterial killing, are also used against helminths. However, at the same time, hookworms have developed an excretory/secretory deoxyribonuclease protein, known as Nb-DNase II, that can degrade the NETs, in both *in vitro* and *in vivo* models. This new finding provides an exciting avenue of targeting parasitic nematode DNAses as vaccine or therapeutic targets to promote NET-mediated nematode killing.

### Extracellular Vesicles

Extracellular vesicles (EVs) released by parasitic nematodes during infection may provide a powerful strategy for the parasitic nematode to generate widespread effects on host cells (Coakley et al., 2015). Of therapeutic promise, treatment with EVs from *T. spiralis* and *N. brasiliensis* suppressed colitis of mice and protection was associated with reduced proinflammatory cytokines and increased Th2 and Treg responses (Eichenberger et al., 2018; Yang et al., 2020). In addition to containing lipids and proteins that may be immunomodulatory, EVs may also serve as cargos to deliver small RNAs to host cells, such as macrophages and intestinal cells, where they target and suppress host RNA. sRNAs have been identified in EVs generated by several parasitic nematodes, including *T. spiralis*, *N. brasiliensis*, *Trichuris muris*, and *H. polygyrus*, where they are predicted to target host immune gene networks (Tritten et al., 2017; Eichenberger et al., 2018; Chow et al., 2019; Yang et al., 2020). For example, *H. polygyrus* EVs were able to suppress macrophage responses and IL-33 signaling, and contained miRNAs that specifically targeted host DUSP1 RNA, a regulator of MAPK signaling (Buck et al., 2014; Coakley et al., 2017). miRNA generation has been reported in several nematode species, including *Ascaris suum*, which infects pigs, where miRNA sequence analysis predicted that they targeted the host Th2 immune response (IL-13, IL-25, IL-33) (Hansen et al., 2019). Circulating filarial nematode-derived miRNAs were detected in the blood of *Litomosomoides sigmodontis*-infected mice, *O. volvulus*-infected humans, *Loa loa*-infected baboons and *Onchocerca ochengi*-infected cattle (Buck et al., 2014; Tritten et al., 2014; Quintana et al., 2015), however, whether they were present in EVs, or targeted host gene expression, is unclear.

The products included in EVs can differ by lifecycle stage and sex of the nematode (Gu et al., 2017; Harischandra et al., 2018). Examining the molecules found in EVs for all lifecycle stages will allow for the discovery of a wide variety of potential drug targets. Molecules found in all developmental stages associated with the host could be strong candidates for the development of vaccines, allowing the immune system to recognize parasites throughout an infection, such as Galectin-2 (Hertz et al., 2020). On the other hand, molecules unique to adults may assist the worm in evading the host immune system in order to maintain a chronic infection, making them of particular interest for the development of anthelminthics and as immunotherapies. Interestingly, adult female *B. malayi* EVs had far more complex proteomes than males, with nearly four times as many proteins, including a MIF homolog, which may be involved in regulating the immune system (Harischandra et al., 2018). EVs may offer containment and protection from degradation of a multitude of immunogenic nematode antigens that could allow for more effective host immune responses to helminths. For example, intact EVs, but not lysed EVs, from *T. muris* were able to reduce egg burden in a subsequent infection of this worm, making them potential vaccine candidates for improved immunogenicity (Coakley et al., 2017; Shears et al., 2018). EVs present a unique opportunity to study the parasitic lifecycle, allowing for a greater understanding of molecules that are required to initiate and maintain an infection.

## Non-Mammalian Model Systems: Entomopathogenic Nematodes and Plant Parasitic Nematodes

Model systems have proven to be valuable to the study of human disease (Rubin et al., 2000). For example, initial analysis of the *Drosophila melanogaster* genome identified that over 60% of human disease-associated genes had orthologs in the fly. Studies of fly immunity led to the discovery and description of the Toll pathway and subsequently Toll-like receptors in mammals (Lemaitre, 2004). Similarly, determining the mechanistic function of conserved parasitic nematode effectors may benefit from the use of the EPN-insect model system, which is cheaper, faster, and allows for more individual hosts to be used per experiment than could possibly be done in a mammalian system. Effectors could be mechanistically described in the model system, providing an elevated starting point for experimentation in parasitic infections of mammals.

EPNs form a mutualistic relationship with bacteria, carrying them inside their intestine when they infect their hosts, releasing the bacteria into the hemolymph of their insect host. The bacteria assist in killing the host, and, along with liquified host tissue, serve as a food source (Bai et al., 2013; Brivio and Mastore, 2018). Similar to skin-penetrating nematode parasites of mammals, the initial infection process is entirely dependent on the ability of the nematode to enter the host and suppress its immune system. EPNs suppress the host immune system early in infection, causing it to tolerate not only nematode parasites but their symbiotic bacteria, until the host succumbs to infection (Toubarro et al., 2013). The specific molecules excreted/secreted from EPNs could be used for pest control in agricultural settings and also for immunoregulatory studies. Here we discuss the current research and known functions of EPN-derived virulence and immunomodulatory molecules, and how they relate to molecules employed by mammalian pathogenic nematodes. We also discuss main virulence factors that are present in plant parasitic nematodes, highlighting the striking conservation of these parasitic virulence mechanisms across the tree of life.

The ES products from the EPN *Heterorhabditis bacteriophora* are lethal to their insect hosts at high concentrations (Kenney et al., 2019). Treatment of *Drosophila* with proteins extracted from the supernatant of activated *H. bacteriophora* suppressed expression of antimicrobial protein diptericin, a product of the immune deficiency (Imd) pathway in insects (Kenney et al., 2019). This immunomodulatory mechanism is swift enough to allow for the infection of not just the nematode, but its mutualistic bacterial co-infector *Photorhabdus luminescens*, which would otherwise be killed by its insect host. In a similar manner, *S. carpocapsae* suppresses the immune response of its *Drosophila* host, allowing for the propagation of the endosymbiotic bacteria *Xenorhabdus nematophila* (Garriga et al., 2020). Shortly after infection, and before the bacteria is released from the gut of the infected nematode, there is a significant reduction in total insect hemocytes, suggesting that the nematode itself is capable of suppressing the host immune system, to the benefit of its endosymbiotic bacteria. The mechanism for immunomodulatory products from EPNs remains to be determined, however it appears to be time sensitive. After 3 h of exposure to live *S. carpocapsae*, insect hemocytes had reduced phagocytic activity, which was not apparent after only 1 h (Brivio et al., 2018). Identification of the specific EPN-derived molecules that target this innate immune Imd pathway in *Drosophila*, and whether they are conserved in mammalian parasitic nematodes could allow discovery of new anthelminthics and immunotherapies.

**FIGURE 2:**
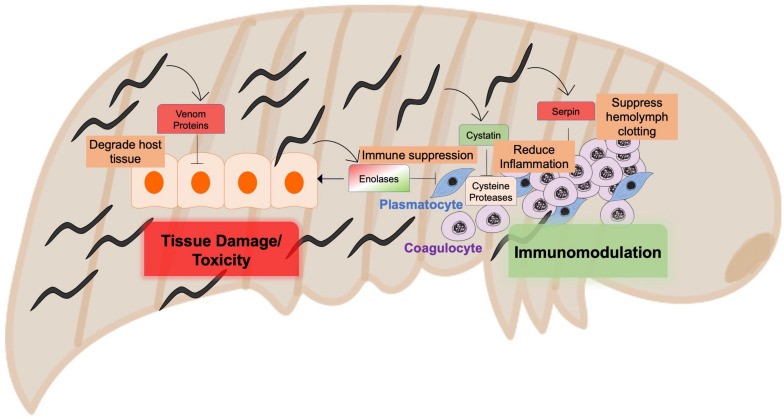
Entomopathogenic nematodes as a model to identify virulent and immunomodulatory nematode-secreted molecules.

There are striking differences between the EPN lifecycle and that of mammalian pathogenic nematodes, most importantly the fate of the host, which is swiftly killed by EPNs in contrast to mammalian parasitic nematodes which establish chronic infections (Cooper and Eleftherianos, 2016). In order to evade the host immune system, contribute to host killing, and then feed on the dead body, the parasite must be able to successfully suppress the host immune response, release toxins, and then digest host tissue, making EPNs a strong model for identifying anti-inflammatory molecules as well as strong virulence factors. Recently, studies characterizing the specific proteins present in EPN ES revealed the remarkable resemblance to mammalian parasitic nematode-derived proteins with regards to structure and function. These include VAL proteins, enolases, serpins, and cystatins (Figure 2). For instance, *Steinernema glaseri* was shown to express enolases only at the activated infectious juveniles (IJs) stage, suggesting that the protein has a role in staging an infection, likely to digest the insect tissue (Liu et al., 2012). In infected insects, this secreted enolase was present in the insect hemolymph, and alone was sufficient to allow for quicker propagation of the bacteria, *Xenorhabdus poinarii*. Many venom proteins, with similarity to mammalian parasitic nematode VAL proteins, were identified in the ES of activated *Steinernema carpocapsae* and *S. feltiae* infective juveniles (IJs), and may contribute to the high toxicity of these parasites to their insect hosts (Lu et al., 2017; Chang et al., 2019). *S. carpocapsae* also expressed the serpin-like inhibitor Sc-SRP-6. Sc-SRP-6 impaired clot formation in its insect host by preventing the incorporation of melanin, known as melanization, which is an important defense mechanism in insects (Toubarro et al., 2013). Likewise, Sc-SRP-6 inhibited the hydrolysis of insect gut juices, a function that is thought to be conserved in *A. ceylanicum*. This serpin-like protein not only modulated the immune system, but inhibited digestion as the nematode passes through the gut of its host. Similar serpin-like genes were also found in mammalian-pathogenic nematodes, such as *B. malayi* (Zang et al., 1999). ES products from EPNs therefore have similar functions to those from mammalian-pathogenic nematodes, and may serve as powerful models for rapid discovery of useful targets for anthelminthics, given the comparative affordability and shorter lifecycles of EPNs. EPNs also produce cystatin, particularly when they detect insect hemolymph, as a location cue for their presence in the insect (Hao et al., 2008). Further research comparing the similarities between cystatins from EPNs and mammalian pathogenic nematodes would be valuable in validating the connections between these models.

Like nematode parasites of animals, plant-parasitic nematodes (PPNs) are masters of immune modulation, most of which is mediated by their secreted proteins and molecules. Because of their devastating effects in agriculture, PPNs are well-studied, and hundreds of secreted effectors have been identified, though, similar to other nematodes, few have been studied in mechanistic detail (Rehman et al., 2016; Vieira and Gleason, 2019). A detailed discussion of PPN effectors is beyond the scope of this review, however several recent reviews focus on PPN virulence factors and host-pathogen interaction (Goverse and Smant, 2014; Rehman et al., 2016; Vieira and Gleason, 2019). Here we discuss PPN virulence factors that are shared with EPN and mammalian parasitic nematodes, including VALs and cystatins. VAL genes have been identified in many PPNs, and their expression is associated with host invasion and migration through host tissues (Lozano-Torres et al., 2014; Wilbers et al., 2018). PPN VALs have been shown to be important for modulating host immunity, especially in the early stages of infection (Kang et al., 2012; Wang et al., 2007). Several VAL-family proteins have been characterized, and they appear to modulate similar processes in both plants and animals, suggesting that mechanistic studies in one model system will be valuable to our understanding of how these effectors work in general. Many cystatin genes have been predicted in plant-parasitic nematode genomes, but little is known about their role in parasitism. A recent description of a cystatin from the pine wood nematodes (PWN) *Bursaphelenchus xylophilus*, found that *Bx-cpi-1* is involved in the development and pathogenic process of the nematode (Xue and Wu, 2019).

The shared ancestry and parasitic behavior of these nematodes could be an explanation for their similar strategies of immune modulation. The conservation of molecular mechanisms of parasitism could allow for the identification of more proteins as well as small molecules that could be used to optimize an immune response during nematode infections, balancing an inflammatory response with worm burden. Further research will allow for ES products to be harnessed for the modulation of the immune system in mammals beyond the context of nematode infections, with applications in allergy and inflammatory diseases, without the risks of infection with live worms. The early stages of EPN infections are of particular interest, as the parasite focuses on suppressing the immune system without killing its host, prior to the release of mutualistic bacteria. This highly immunosuppressive stage may have applications with the mammal-infecting parasites that persist in their hosts for months or even years, such as hookworms and filarial nematodes (Hotez et al., 2008).

## Conclusion

Here we discussed some of the main nematode-derived effectors in virulence and host immunomodulation. With improved high throughput technologies, further research is being conducted to identify more specific molecules that are involved in host-parasite interaction, that could provide therapeutic insight for controlling helminth infections, and at the same time curing debilitating inflammatory diseases. We also discussed EPNs and PPNs as cheaper and faster models for understanding how nematode-derived molecules influence host-pathogen interactions. The combined strategies from screening for novel nematode-derived molecules, to determining their therapeutic abilities by mechanistic studies in vertebrate, invertebrate, and plant models, and finally testing them in clinical studies will allow for faster development of anthelminthic drugs, vaccines, and therapies for allergy and inflammatory disease.

## Author Contributions

MN and SB contributed writing as well as the organization and idea to the work. AD contributed significant writing. All authors have approved it for publication.

## Conflict of Interest

The authors declare that the research was conducted in the absence of any commercial or financial relationships that could be construed as a potential conflict of interest.
